# The resuscitation-promoting factors of *Mycobacterium tuberculosis* are required for virulence and resuscitation from dormancy but are collectively dispensable for growth *in vitro*

**DOI:** 10.1111/j.1365-2958.2007.06078.x

**Published:** 2007-12-21

**Authors:** Bavesh D Kana, Bhavna G Gordhan, Katrina J Downing, Nackmoon Sung, Galina Vostroktunova, Edith E Machowski, Liana Tsenova, Michael Young, Arseny Kaprelyants, Gilla Kaplan, Valerie Mizrahi

**Affiliations:** 1 MRC/NHLS/WITS Molecular Mycobacteriology Research Unit, DST/NRF Centre of Excellence for Biomedical TB Research, School of Pathology, University of the Witwatersrand and the National Health Laboratory Service Johannesburg 2000, South Africa.; 2 Laboratory of Mycobacterial Immunity and Pathogenesis, Public Health Research Institute, International Center for Public Health 225 Warren St., Newark, NJ 07103-3535, USA.; 3 Bakh Institute of Biochemistry Leninsky pr.33, 119071 Moscow, Russia.; 4 Institute of Biological Sciences, Aberystwyth University Ceredigion SY23 3DD, Wales, UK.

## Abstract

*Mycobacterium tuberculosis* contains five resuscitation-promoting factor (Rpf)-like proteins, RpfA-E, that are implicated in resuscitation of this organism from dormancy via a mechanism involving hydrolysis of the peptidoglycan by Rpfs and partnering proteins. In this study, the *rpfA-E* genes were shown to be collectively dispensable for growth of *M. tuberculosis* in broth culture. The defect in resuscitation of multiple mutants from a ‘non-culturable’ state induced by starvation under anoxia was reversed by genetic complementation or addition of culture filtrate from wild-type organisms confirming that the phenotype was associated with *rpf*-like gene loss and that the ‘non-culturable’ cells of the mutant strains were viable. Other phenotypes uncovered by sequential deletion mutagenesis revealed a functional differentiation within this protein family. The quintuple mutant and its parent that retained only *rpfD* displayed delayed colony formation and hypersensitivity to detergent, effects not observed for mutants retaining only *rpfE* or *rpfB*. Furthermore, mutants retaining *rpfD* or *rpfE* were highly attenuated for growth in mice with the latter persisting better than the former in late-stage infection. In conjunction, these results are indicative of a hierarchy in terms of function and/or potency with the Rpf family, with RpfB and RpfE ranking above RpfD.

## Introduction

*Mycobacterium tuberculosis* is an exquisitely adapted human pathogen that has infected one-third of the world's population (Dye *et al*., 2002) and is responsible for approximately eight million new cases of tuberculosis (TB) and two million deaths each year (Corbett *et al*., 2003). *M. tuberculosis* can persist in the host for decades after infection before reactivating to cause disease (Stewart *et al*., 2003). The bacterial and host factors that contribute towards latent TB infection (LTBI) and reactivation disease have long remained enigmatic. However, there is considerable circumstantial evidence to suggest that the persisting organisms may include bacteria in physiological states that are characterized by impaired culturability (i.e. colony-forming ability) (Mukamolova *et al*., 2003; Young *et al*., 2005). These observations suggest a plausible link between an intrinsic microbiological property of *M. tuberculosis*– the ability to enter into a state of dormancy from which culturability can be restored – and the clinically defined phenomenon of LTBI.

Resuscitation-promoting factor (Rpf) from *Micrococcus luteus* is the founder member of a family of secreted proteins found throughout the actinobacteria (Mukamolova *et al*., 1998; Kell and Young, 2000). Rpf is able to restore culturability from a dormant state and also stimulate growth of viable bacteria, acting at concentrations of pM or less (Mukamolova *et al*., 1998; 2002a). *Mi. luteus* Rpf was recently shown to possess muralytic activity (Mukamolova *et al*., 2006), consistent with its structural similarity to C-type lysozyme and soluble lytic transglycosylases (Cohen-Gonsaud *et al*., 2005). The decline in both peptidoglycan hydrolysis and resuscitating-promoting activities of Rpf caused by mutation of a predicted active site glutamic acid residue strongly implicated this protein in cell wall remodelling at the level of the peptidoglycan (Mukamolova *et al*., 2006). However, while several possibilities have been proposed (Ravagnani *et al*., 2005; Keep *et al*., 2006), the precise mechanism by which Rpf-catalysed hydrolysis of the peptidoglycan promotes bacterial resuscitation has yet to be elucidated.

*Mycobacterium tuberculosis* possesses five *rpf* homologues, *rpfA-E* (Mukamolova *et al*., 2002b), all of which are expressed *in vitro* and in mice (Tufariello *et al*., 2004). Expression of some of these genes has also been observed in human TB infection (Fenhalls *et al*., 2002; Rachman *et al*., 2006). Recombinant forms of RpfA-E show similar biological activity to the *Mi. luteus* protein (Mukamolova *et al*., 2002b). Individually, the *rpfA-E* genes are dispensable for growth of *M. tuberculosis in vitro* and *in vivo* (Downing *et al*., 2004; 2005; Tufariello *et al*., 2004), suggesting functional redundancy. However, more recent evidence has suggested some degree of functional specialization within this gene family: Tufariello *et al*. found that an *rpfB-*defective mutant of *M. tuberculosis* Erdman displayed delayed kinetics of reactivation in a mouse model of dormancy (Tufariello *et al*., 2006) and we found that two mutants of *M. tuberculosis* H37Rv lacking three *rpf*-like genes in different combinations were impaired for resuscitation from a ‘non-culturable’ state and were differentially attenuated for growth in mice (Downing *et al*., 2005). In an important new advance, RpfB was shown to interact with a putative mycobacterial endopeptidase, designated as Rpf-interacting protein A (RipA) (Hett *et al*., 2007). The two proteins colocalize to the septa of dividing cells suggesting a role for the RipA–RpfB complex in peptidoglycan hydrolysis during cell division. RipA also interacts with RpfE but not with RpfA, RpfC or RpfD, suggesting that these Rpfs may act via distinct mechanisms and/or on different peptidoglycan substrates, possibly in conjunction with other RipA-like proteins (Hett *et al*., 2007). To further investigate the individual and collective roles of the Rpfs in *M. tuberculosis*, we constructed three quadruple mutants and a quintuple mutant of H37Rv lacking all five genes, and in this paper, we demonstrate that although the *rpf*-like genes are required for virulence and resuscitation from dormancy, the entire *rpfA-E* family is dispensable for growth of this organism *in vitro*.

## Results

### The *rpfA-E* genes are collectively dispensable for growth of *M. tuberculosis* in broth culture

**Fig. 1:**
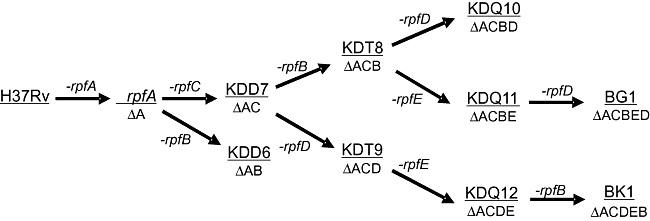
Stepwise deletion of *rpf*A-E genes in *M. tuberculosis* H37Rv. The arrows represent in-frame deletions introduced by allelic exchange mutagenesis to produce the strains whose names are underlined. For the sake of simplicity, the mutant strains are referred to throughout the text according to their abbreviated genotypes, which are given beneath the strain names and are named according to the order in which the *rpf*-like genes were deleted.

The strategy for sequential deletion of the five *rpf*-like genes in *M. tuberculosis* is shown in Fig. 1 with allelic exchange mutagenesis resulting in unmarked in-frame deletions (Figs S1 and S2), as previously described (Downing *et al*., 2004; 2005). The quadruple mutants, ΔACBD, ΔACBE, ΔACDE, and the quintuple mutant, ΔACBED, were tested for their ability to grow in broth culture and survive in aerobic stationary phase. Like the progenitor triple mutants from which they were derived (Downing *et al*., 2005), no growth defects were observed for any of the strains tested; all displayed approximately the same doubling times during logarithmic growth and achieved similar cell densities in stationary phase (data not shown). Moreover, no loss of culturability was observed during maintenance in stationary phase over a period of 10 days, as determined by colony-forming unit (cfu) enumeration (data not shown).

### Effect of progressive *rpf*-like gene loss on expression of the remaining *rpf*-like genes

To evaluate the effect of progressive *rpf*-like gene loss on expression of the remaining *rpf*-like genes, the steady-state, logarithmic-phase expression levels of the *rpf*-like genes remaining in the double, triple and quadruple mutants were compared with those observed in the wild-type control by real-time, quantitative reverse transcription-PCR (qRT-PCR) analysis (Table 1). Previous work had revealed that *rpf*-like gene loss was associated with upregulation of the remaining *rpf*-like genes, with the exception of *rpfA*, which was downregulated (Downing *et al*., 2004). In the present study, some subtle changes in steady-state expression levels were observed. For example, *rpfB* was significantly upregulated in ΔAC, but downregulated in ΔACDE and *rpfD* was upregulated in ΔAB but downregulated in ΔACBE. Overall, the elevated expression of *rpfB-E* observed in the single mutant strains was negated in the multiple mutants: *rpfB*, *rpfD* and *rpfE* all showed a similar trend towards reduced expression with progressive *rpf*-like gene loss, being expressed up to twofold higher than wild-type levels in the double and triple mutants, but declining to 55–75% of wild-type levels in the quadruple mutants.

**Table 1:** Expression analysis by qRT-PCR of remaining *rpf*-like genes in the multiple *rpf*-like mutant strains.

		*rpf*-like gene expression in the mutant strains relative to H37Rv			
		
Strain	Mutant genotype	rpfB	rpfC	rpfD	rpfE
H37Rv	Wild-type	1.00	1.00	1.00	1.00
KDQ10	ΔACBD	–	–	–	0.6 ± 0.18**
KDQ11	ΔACBE	–	–	0.55 ± 0.26*	–
KDQ12	ΔACDE	0.75 ± 0.02*	–	–	–
KDT8	ΔACB	–	–	1.02 ± 0.33	2.01 ± 0.53**
KDT9	ΔACD	1.21 ± 0.38	–	–	1.89 ± 0.34**
KDD6	ΔAB	–	1.25 ± 0.57	1.66 ± 0.30***	1.78 ± 0.14***
KDD7	ΔAC	1.53 ± 0.23***	–	1.42 ± 0.43	2.17 ± 0.79**

* *P* < 0.1

** *P* < 0.05

*** *P* < 0.01.

### Progressive *rpf*-like gene loss differentially affects colony formation of *M. tuberculosis* on agar-solidified media

**Fig. 2:**
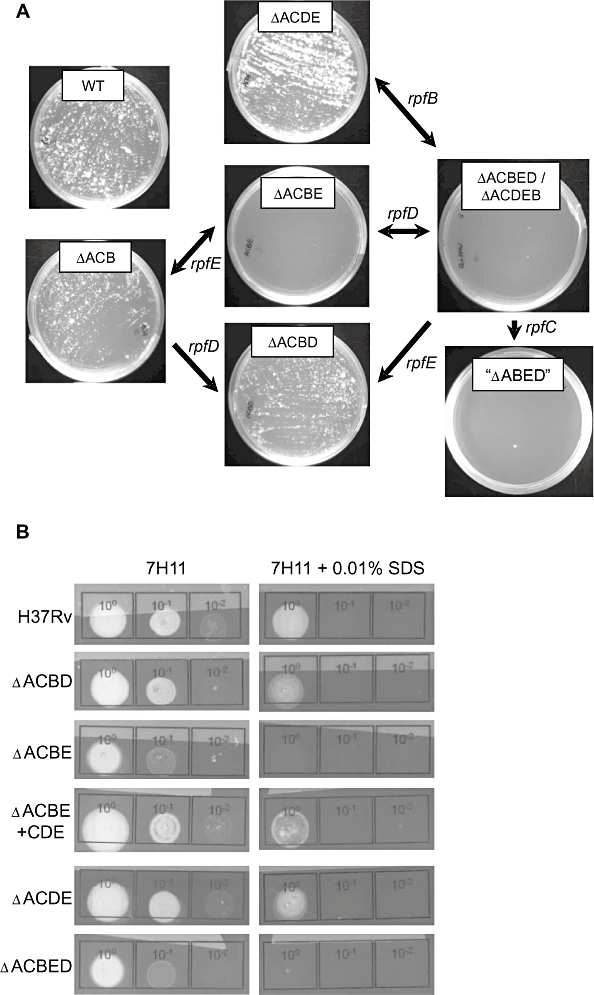
Delayed colony formation and SDS hypersensitivity of Rpf-deficient mutants of *M. tuberculosis*. A. Log-phase cultures of selected strains were serially diluted and plated onto Middlebrook 7H11 agar. Plates were incubated for 18 days before scoring for growth. Deletions and/or complementation of specific *rpf*-like genes are shown by single or double-headed arrows. B. Serial dilutions of log-phase cultures of selected strains were spotted on 7H11 agar with or without SDS at a concentration of 0.01%. Plates were incubated for 10 days before scoring for growth. The relatively poor growth of the ΔACBE and ΔACBED strains on 7H11 agar is due to their delayed colony formation on this medium (A).

In contrast to the normal growth observed in broth culture, the quadruple mutant retaining *rpfD*, ΔACBE and its quintuple mutant derivative, ΔACBED, both displayed a marked plating phenotype, as evidenced by delayed colony formation on Middlebrook 7H11 agar (Fig. 2A and data not shown). Interestingly, the delay in colony formation observed for ΔACBED and its derivative carrying the empty integration vector used for genetic complementation (pMV306H) was no worse than that of ΔACBE (Fig. 2A and data not shown). Plates inoculated with ΔACBE, ΔACBED or ΔACBED carrying pMV306H required 34 days' incubation for the average colony size to reach that achieved by the wild-type strain in 18 days (data not shown). ΔACBE and ΔACBED also showed delayed colony formation on 7H10 agar, although the effect was less pronounced (data not shown). In contrast, the *rpfE*-containing quadruple mutant, ΔACBD, and the *rpfB*-containing quadruple mutant, ΔACDE, showed kinetics of colony formation on 7H11 agar that were similar to those of the wild-type strain (Fig. 2A).

To investigate the association between the delayed colony formation of ΔACBE and ΔACBED and *rpf*-like gene loss, a series of integration vectors carrying one or more of the deleted genes with their putative promoter regions were constructed and introduced into the mutant strains (Table 2 and Fig. S3). We attempted to reverse-engineer two of the quadruple mutants to simpler genotypes by transformation with vectors carrying multiple *rpf*-like genes. ΔACBD was thus transformed with a construct carrying *rpfC*, *rpfB* and *rpfD* to ‘re-create’ the ΔA genotype. A similar approach was attempted with ΔACBE but vectors carrying combinations of *rpfC*, *rpfB* and *rpfE* were unstable in *Escherichia coli* (data not shown). Therefore, this mutant was transformed instead with a vector carrying *rpfC*, *rpfD* and *rpfE* to generate a strain with a genotype analogous to ΔAB, but carrying an additional copy of *rpfD*.

**Table 2:** Strains and plasmids used in this study.

Name	Description	Source/reference
Strains		
*E. coli*		
DH5α	Strain used for routine cloning; *supE44*Δ*lacU169 (*φ*80 lacZ*Δ*M15*) *hsdR17 recA1 endA1 gyrA96 thi-1 relA1*	Laboratory stock
*M. tuberculosis*^a^		
H37Rv	Virulent reference laboratory strain (ATCC 25618)	Laboratory stock
KDD6 (ΔAB)	Double mutant of *M. tuberculosis* H37Rv carrying internal in-frame deletions in *rpfA* and *rpfB*; Δ*rpfA*Δ*rpfB*	Downing *et al*. (2005)
KDD7 (ΔAC)	Double mutant of *M. tuberculosis* H37Rv carrying internal in-frame deletions in *rpfA* and *rpfC*; Δ*rpfA*Δ*rpfC*	Downing *et al*. (2005)
KDT8 (ΔACB)	Triple *rpf*-like mutant; derivative of KDD7 carrying internal in-frame deletion in *rpfB*; Δ*rpfA*Δ*rpfC*Δ*rpfB*	Downing *et al*. (2005)
KDT9 (ΔACD)	Triple *rpf-*like mutant; derivative of KDD7 carrying internal in-frame deletion in *rpfD*; Δ*rpfA*Δ*rpfC*Δ*rpfD*	Downing *et al*. (2005)
KDT8::pHRPFCB (ΔACB + CB)	KDT8 carrying the *rpfC* and *rpfB* genes in pHRPFCB and integrated at the *attB* locus; Hyg^R^	This work
KDT9::pHRPFCD (ΔACD + CD)	KDT9 carrying the *rpfC* and *rpfD* genes in pHRPFCD and integrated at the *attB* locus; Hyg^R^	This work
KDQ10 (ΔACBD)	Quadruple *rpf*-like mutant; derivative of KDT8 carrying internal in-frame deletions in *rpfD*; Δ*rpfA*Δ*rpfC*Δ*rpfB*Δ*rpfD*	This work
KDQ11 (ΔACBE)	Quadruple *rpf*-like mutant; derivative of KDT8 carrying internal in-frame deletion in *rpfE*; Δ*rpfA*Δ*rpfC*Δ*rpfB*Δ*rpfE*	This work
KDQ12 (ΔACDE)	Quadruple *rpf*-like mutant; derivative of KDT9 carrying internal in-frame deletion in *rpfE*; Δ*rpfA*Δ*rpfC*Δ*rpfD*Δ*rpfE*	This work
BG1 (ΔACBED)	Quintuple *rpf-*like mutant; derivative of KDQ11 carrying internal in-frame deletion *in rpfD*; Δ*rpfA*Δ*rpfC*Δ*rpfB*Δ*rpfE*Δ*rpfD*	This work
BK1 (ΔACDEB)	Quintuple *rpf-*like mutant; derivative of KDQ12 carrying internal in-frame deletion *in rpfB*; Δ*rpfA*Δ*rpfC*Δ*rpfD ΔrpfE*Δ*rpfB*	This work
KDQ10::pHRPFCBD (ΔACBD + CBD)	KDQ10 carrying *rpfC*, *rpfB* and *rpfD* in pHRPFCDE and integrated at the *attB* locus; Hyg^R^	This work
KDQ11::pHRPFCDE (ΔACBE + CDE)	KDQ11 carrying *rpfC*, *rpfD* and *rpfE* in pHRPFCDE and integrated at the *attB* locus; Hyg^R^	This work
KDQ11::pMRPFE (ΔACBE + E)	KDQ11 carrying *rpfE* cloned in pMRPFE and integrated at the *attB* locus; Hyg^R^	This work
KDQ11::pMLUWP (ΔACBE::pMLUWP)	KDQ11 carrying *Mi. luteus rpf* gene cloned in pMLUWP and integrated at the *attB* locus; Hyg^R^	This work
BG1::pMV306H (ΔACBED::pMV306H)	BG1 carrying pMV306H integrated at the *attB* locus; Hyg^R^	This work
BG1::pMRPFB (ΔACBED + B)	BG1 carrying *rpfB* in pMRPFB and integrated at the *attB* locus; Hyg^R^	This work
BG1::pHRPFC (ΔACBED + C)	BG1 carrying *rpfC* in pHRPFC and integrated at the *attB* locus; Hyg^R^	This work
BG1::pMRPFD (ΔACBED + D)	BG1 carrying *rpfD* in pMRPFD and integrated at the *attB* locus; Hyg^R^	This work
BG1::pMRPFE (ΔACBED + E)	BG1 carrying *rpfE* in pMRPFE and integrated at the *attB* locus; Hyg^R^	This work
Plasmids		
pHINT	*E. coli*– Mycobacterium integrating shuttle vector; Ap^R^, Hyg^R^	O'Gaora *et al*. (1997)
pMV306H	*E. coli –* Mycobacterium integrating shuttle vector; derivative of pMV306 (Stover *et al*., 1991) carrying a *hyg* gene; Hyg^R^	H. Boshoff
pBluescriptKS+	*E. coli* vector for cloning PCR products; Ap^R^	Promega
pSMT3RPF Hyg^R^	Derivative of pSMT3 (O'Gaora *et al*., 1997) carrying *Mi. luteus rpf* under control of the mycobacterial *hsp60* promoter;	G. Mukamolova
pGS3RPF	Derivative of pGINT carrying *Mi. luteus rpf* under control of the mycobacterial *hsp60* promoter, Gm^R^	This work
pMLUWP	Derivative of pHINT carrying *Mi. luteus rpf* under control of the mycobacterial *hsp60* promoter, Hyg^R^	This work
pEM75	Derivative of pBluescriptKS+ with *bla* gene replaced by *aph* cassette; Km^R^	This work
pRPFBΔ2	Knockout vector for constructing internal in-frame deletion mutation in *rpfB*; Hyg^R^, Km^R^	Downing *et al*. (2004)
pRPFDΔ2	Knockout vector for constructing internal in-frame deletion mutation in *rpfD*; Hyg^R^, Km^R^	Downing *et al*. (2004)
pRPFEΔ2	Knockout vector for constructing internal in-frame deletion mutation in *rpfE*; Hyg^R^, Km^R^	Downing *et al*. (2004)
pEMRPFC	Derivative of pEM75 carrying 4249 bp HindIII-Asp718 fragment containing *rpfC*; Km^R^	This work
pHRPFC	Derivative of pHINT carrying *rpfC*; Hyg^R^	This work
pEMRPFB	Derivative of pEM75 carrying 3027 bp XhoI fragment containing *rpfB*; Km^R^	This work
pEMRPFB2	Derivative of pEM75 carrying 6152 bp BamHI-HindIII fragment containing *rpfB;* Km^R^	This work
pBRPFD	Derivative of pBluescriptKS+ carrying 1656 bp PCR product containing *rpfD*; Ap^R^	This work
pBRPFE	Derivative of pBluescriptKS+ carrying 1180 bp PCR product containing *rpfE*, Ap^R^	This work
pHRPFCD	Derivative of pHINT carrying *rpfC* and *rpfD*; Hyg^R^	This work
pHRPFCDE	Derivative of pHINT carrying *rpfC*, *rpfD* and *rpfE*; Hyg^R^	This work
pHRPFCB	Derivative of pHINT carrying *rpfC* and *rpfB*; Hyg^R^	This work
pHRPFCBD	Derivative of pHINT carrying *rpfC*, *rpfB* and *rpfD*; Hyg^R^	This work
pMRPFB	Derivative of pMV306H carrying *rpfB*; Hyg^R^	This work
pMRPFD	Derivative of pMV306H carrying *rpfD*; Hyg^R^	This work
pMRPFE	Derivative of pMV306H carrying *rpfE*; Hyg^R^	This work

a The abbreviated, genotype-based nomenclature is given in brackets after the strain name.

Ap^R^, ampicillin-resistant; Gm^R^, gentamicin-resistant; Hyg^R^, hygromycin-resistant; Km^R^, kanamycin-resistant.

Expression of *rpfA-E* in the mutant strains and complemented derivatives was analysed by RT-PCR (Fig. S4). All of the mutant strains showed *rpf*-like gene expression profiles consistent with their genotypes. As expected, *rpfA* transcript was not detected in any of the strains (data not shown). *rpfB* transcript was not detected when carried on pHRPFCB in ΔACB (Fig. S4, lane 8) or on pHRPFCBD in ΔACBD (Fig. S4, lane 15) but this gene was expressed in ΔACBED when cloned on a different fragment in pMRPFB (Fig. S4, lane 18). *rpfC*, *rpfD* and *rpfE* were expressed from all complementation vectors, whether cloned alone or in combination with other genes.

The delayed colony formation of ΔACBED was not affected by complementation with *rpfC* or *rpfD* but was corrected by the introduction of either *rpfB* or *rpfE* (Fig. 2A). These phenotypes were consistent with reconstruction of the ΔACBE, ΔACBD or ΔACDE genotypes by integration of *rpfD*, *rpfE* or *rpfB*, respectively, in ΔACBED. The phenotype of ΔACBED carrying pHRPFC suggested that a quadruple mutant with a Δ*rpfA*Δ*rpfB*Δ*rpfD*Δ*rpfE* genotype (not made in this study) would display the same delay in colony formation as ΔACBE and ΔACBED. Complementation of ΔACBE with *rpfE* also completely reversed its delayed colony-forming phenotype, consistent with reconstruction of the genotype of ΔACB, which shows no delay in colony formation (Fig. 2A). In contrast, the delayed colony-forming phenotype of ΔACBE was only partially corrected by complementation with the *rpf* from *Mi. luteus* suggesting that *M. tuberculosis* RpfE was functionally superior in this regard to the non-cognate Rpf (data not shown).

### Mutants lacking *rpfB* and *rpfE* are hypersensitive to sodium dodecylsulphate

As mentioned above, the delayed colony formation of ΔACBE and ΔACBED was more pronounced on Middlebrook 7H11 than 7H10 agar. A key difference between these media is the fourfold higher concentration of malachite green in the former. As malachite green sensitivity is suggestive of a cell wall defect (Gillespie *et al*., 1986), the effects of Rpf deficiency on sensitivity to a cell wall damaging agent were assessed. The susceptibility of the quadruple and quintuple mutant strains to the detergent, sodium dodecylsulphate (SDS), was compared with that of the wild-type by spotting serial dilutions of logarithmic-phase cultures on 7H11 agar containing 0.001–0.1% SDS (Fig. 2B and data not shown). No growth inhibition was observed for the wild-type or any of the mutant strains at an SDS concentration of 0.001%, whereas 0.1% SDS was severely growth inhibitory to all strains (not shown). However, differential sensitivities were observed at an SDS concentration of 0.01% (Fig. 2B). Under these conditions, the SDS sensitivity of the mutants retaining *rpfE* (ΔACBD) or *rpfB* (ΔACDE) was similar to that of the wild-type strain. In contrast, the quadruple and quintuple mutants lacking both of these genes (ΔACBE and ΔACBED) displayed marked hypersensitivity to SDS (Fig. 2B). Importantly, the phenotype of the ΔACBE mutant was partially reversed by complementation with *rpfC*, *rpfD* and *rpfE*, confirming its association with Rpf deficiency (Fig. 2B).

### Resuscitation of *rpf*-deficient mutants from a ‘non-culturable’ state

The triple mutants, ΔACB and ΔACD, were previously shown to be unable to resuscitate spontaneously from a state of non-culturability induced by starvation without oxygen input using a most probable number (MPN) assay (Downing *et al*., 2005). To investigate the association between the resuscitation defect and *rpf*-like gene loss, we assessed ΔACB, ΔACD, ΔACBE and ΔACBD and their complemented derivatives in this model alongside a wild-type control (Table 3). As expected, H37Rv resuscitated spontaneously from a ‘non-culturable’ state (Downing *et al*., 2005), with its resuscitation being further stimulated by the addition of culture filtrate from an actively growing culture of wild-type *M. tuberculosis* (Shleeva *et al*., 2002). As previously observed, both triple mutants were also defective for resuscitation in this model (Downing *et al*., 2005). However, this defect was corrected in ΔACB and ΔACD both by the addition of culture filtrate from the wild-type strain and by genetic complementation (Table 3).

**Table 3:** Resuscitation of dormant (non-culturable) cultures of *rpf*-like mutants and complemented derivatives.

			MPN per ml			MPN per ml (+ CF)		
				
				95% confidence limits			95% confidence limits	
						
Strain	Genotype^a^	cfu per ml	MPN	MPN	Lower	Higher	Lower	Higher
H37Rv	Wild-type	0	1.8 × 10^7^	5.4 × 10^6^	6 × 10^7^	5.8 × 10^8^	1.8 × 10^8^	1.9 × 10^9^
KDT8	ΔACB	0	0	–	–	1 × 10^3^	0.3 × 10^3^	3.3 × 10^3^
KDT8::pHRPFCB	ΔACB + CB	0	1.2 × 10^5^	3.6 × 10^4^	4 × 10^5^	4.4 × 10^6^	1.3 × 10^6^	1.5 × 10^7^
KDT9	ΔACD	0	0	–	–	2.2 × 10^3^	0.7 × 10^3^	7.7 × 10^3^
KDT9::pHRPFCD	ΔACD + CD	0	4 × 10^4^	1.2 × 10^4^	1.2 × 10^5^	1.2 × 10^5^	3.6 × 10^4^	4 × 10^5^
KDQ10	ΔACBD	0	0	–	–	> 2.4 × 10^4^	7 × 10^3^	–b
KDQ10::pHRPFCBD	ΔACBD + CBD	0	1.2 × 10^5^	3.6 × 10^4^	4 × 10^5^	1.2 × 10^5^	3.6 × 10^4^	4 × 10^5^
KDQ11	ΔACBE	0	0	–	–	> 2.4 × 10^4^	7 × 10^3^	–b
KDQ11::pHRPFCDE^c^	ΔACBE + CDE	1 × 10^5^	> 5 × 10^8^	1.5 × 10^8^	–c	> 5 × 10^7^	1.5 × 10^7^	–b

a Genotypes are shown using the abbreviated nomenclature given in Table 2 and Fig. S4.

b Upper limit was not tested.

c This sample was not rendered fully dormant (non-culturable) in the Sauton's medium/sealed flask model.

CF, culture filtrate.

Like their parental ΔACB strain (Downing *et al*., 2005), both ΔACBD and ΔACBE were resuscitation-defective (Table 3). Complementation of ΔACBD with pHRPFCBD partly rescued this phenotype, as evidenced by the increase in MPN value from 0 to 1.2 × 10^5^ per ml. A complemented derivative of ΔACBE was also included in this analysis, but its phenotype could not be assessed owing to the high background of culturable bacilli in this sample at the end of the 3.5 month starvation period. However, supplementation of the resuscitation medium with culture filtrate resulted in spontaneous resuscitation of both quadruple mutants, as evidenced by the increase in MPN values from 0 to > 2.4 × 10^4^ per ml.

### Rpf deficiency attenuates the growth and persistence of *ΔACBD* and *ΔACBE* in mice

**Fig. 3:**
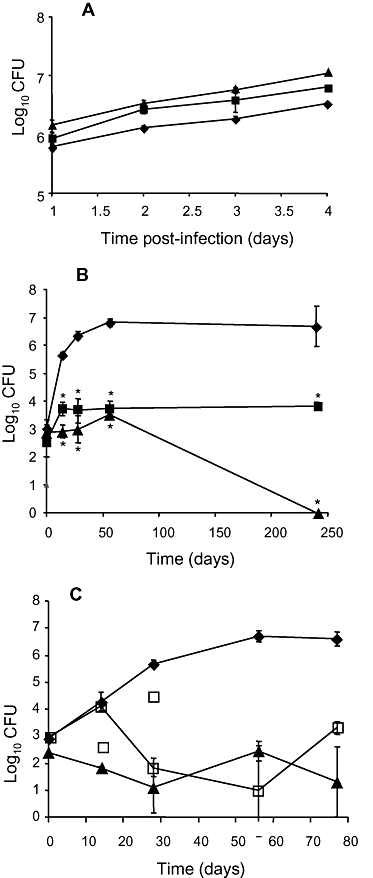
Growth and survival of multiple *rpf*-like mutants in human monocytes and in B6D2/F_1_ mice. A. Human monocytes were infected with H37Rv (♦), ΔACBD (▪) or ΔACBE (▴) and growth was monitored over 4 days. Experiments were conducted with cells isolated from two healthy donors. B. Growth and survival of quadruple mutants in mouse lungs. B6D2/F_1_ mice were infected with H37Rv (♦), ΔACBD (▪) or ΔACBE (▴) and the bacillary loads in the lungs of the infected animals were determined by cfu assessment over a period of 240 days. Each point represents the mean of three mice per group and the error bars denote the standard deviations. The lung bacillary loads that differ significantly from those of the wild-type control are denoted by an asterisk above the relevant data point (*P* < 0.0001 for all points except ΔACBD at 240 days, where *P* = 0.0024). C. Effect of complementation of ΔACBE with *rpfC*, *rpfD* and *rpfE* genes on growth in mouse lungs. B6D2/F_1_ mice were infected with H37Rv (♦), ΔACBE (▴) or ΔACBE + CDE (□) and lung bacillary loads were assessed over 77 days. The individual bacillary loads or the mean of two or three infected mice per group are shown.

To assess the effect of *rpf*-like gene loss on intracellular growth of *M. tuberculosis*, human peripheral blood mononuclear cells (PBMCs) were infected with ΔACBD, ΔACBE or H37Rv and growth monitored over 4 days. Both quadruple mutants grew similarly to the wild-type strain in this model (Fig. 3A). Moreover, both strains retained their respective colony-forming abilities following passage in monocytes: normal for ΔACBD and retarded in the case of ΔACBE (data not shown).

The ΔACBD and ΔACBE strains were then assessed for growth in a murine infection model. B6D2/F_1_ mice were infected through the aerosol route to seed 300–1500 bacteria in the lungs (Fig. 3B). The progress of infection was monitored by scoring the lungs and spleen for cfu over 240 days and monitoring a group of mice infected with each strain for survival. In contrast to the wild-type, which grew by almost 4 logs over 56 days, the quadruple mutants showed significantly reduced growth over this period (*P* < 0.0001 at 56 days). Although the lung bacillary load of ΔACBD persisted at ∼6300 cfu over a period of 240 days, the number of bacteria remaining in the lungs of ΔACBE-infected mice at 240 days was below the limit of detection (40 cfu per lung). Both quadruple mutants were defective for dissemination to the spleen with ΔACBD achieving a final bacterial load *ca*. 100-fold lower than the wild-type and ΔACBE being below the limit of detection by the end of the experiment (data not shown). At 240 days, nine out of 10 of the mice infected with the wild-type strain had succumbed to the infection whereas none of the mice infected with either mutant had died, consistent with the profound attenuation of these strains.

To establish whether the attenuation of the ΔACBE mutant was attributable specifically to *rpf*-like gene loss, the *rpfC*, *rpfD* and *rpfE* genes were integrated at the *attB* site of this mutant via pHRPFCDE. RT-PCR analysis of steady-state levels of mRNA confirmed that the vector-borne *rpfC* and *rpfE* genes were expressed in the complemented mutant (Fig. S4, lane 16) implying that its genotype was analogous to that of the ΔAB strain, albeit with an additional copy of *rpfD*. Although the phenotype of ΔAB had not been assessed *in vivo*, the trend towards increased attenuation with progressive *rpf*-like gene loss observed previously (Downing *et al*., 2005) suggested that restoration of *rpfC* and *rpfE* function in the ΔACBE mutant would be expected to result in an increase in virulence. B6D2/F_1_ mice were thus infected with ΔACBE, the complemented strain or H37Rv and lung cfu scored for groups of three mice over 77 days (Fig. 3C). Growth of the wild-type strain was comparable with that observed in previous infections (Fig. 3B and data not shown). ΔACBE showed no detectable growth in mouse lung over the 77 day time-course and at day 14, mice infected with this strain showed a 200-fold lower bacillary load compared with those infected with the wild-type, consistent with previous observations (Fig. 3B). By comparison, a 40-fold increase in cfu was observed at day 14 in two mice infected with the complemented strain, but no increase was observed in the third animal (shown as an outlier in Fig. 3C). Similarly, at day 28, one animal had a higher bacillary load than the other two, which were at levels comparable to ΔACBE. However, at later time points, both mutant and complemented strains behaved similarly in all animals tested, showing ≥ 1000-fold lower cfu in the lung compared with the wild-type. Furthermore, the lungs of mice sacrificed at the later points showed no significant difference in gross pathology between those infected with the mutant versus complemented strains, with no visible signs of disease being evident. In contrast, the lungs of mice infected with the wild-type strain were enlarged and completely covered with granulomatous lesions, as expected for chronic infection with virulent *M. tuberculosis* (data not shown).

### Analysis of phthiocerol dimycocerosate production by the Rpf-deficient mutants

Spontaneous mutations at the phthiocerol dimycocerosate (PDIM) locus which affect the ability of *M. tuberculosis* to produce this complex lipid have been reported to occur frequently (Manjunatha *et al*., 2006). Loss of PDIM has been associated with attenuation in mice during early (Camacho *et al*., 1999; Cox *et al*., 1999) and chronic infection (Rousseau *et al*., 2004). Given the equivocal results obtained from the virulence assessment of the complemented ΔACBE mutant, we investigated whether mutations affecting PDIM production might account, at least in part, for the attenuation observed for the multiple *rpf*-like mutants observed in this and prior work (Downing *et al*., 2005). The wild-type, quadruple and quintuple mutant strains were tested for PDIM production by monitoring the incorporation of [^14^C]-propionate into lipid. All strains, including the original laboratory stock of H37Rv that was used to produce the ΔA parent from which all multiple mutants were derived (Downing *et al*., 2004) (Fig. 1), and the subclone thereof that was used for all mouse infections, were equally impaired for PDIM production and resembled a mutant of *M. tuberculosis* rendered PDIM-deficient by deletion of the *mmpL7* gene (Domenech *et al*., 2005) (Fig. S5). These results suggested that the attenuation of the *rpf* mutants could not be explained by loss of PDIM.

## Discussion

The multiplicity of *rpf*-like genes in *M. tuberculosis* has presented major challenges for dissecting out the roles of the individual genes and their encoded proteins in growth and culturability, their relationship to one another, and the mechanisms of regulation of their expression and activity. We and others showed previously that each of the *rpfA-E* genes was individually dispensable for growth *in vitro* and *in vivo* (Downing *et al*., 2004; Tufariello *et al*., 2004). We subsequently demonstrated that two triple mutants were defective for resuscitation *in vitro* and were significantly yet differentially attenuated for growth *in vivo* (Downing *et al*., 2005). However, key questions remained, many of which have been addressed in the present study. We have now shown that the entire *rpfA-E* gene family is dispensable for *in vitro* growth. Importantly, quintuple mutants lacking all five genes were obtained independently from two distinct lineages of mutants (Fig. 1 and Table 2). To our knowledge, mutant strains of *M. tuberculosis* carrying this number of unmarked deletion mutations have not been reported previously in the literature. The dispensability of RpfA-E for growth of *M. tuberculosis* H37Rv was somewhat surprising given the inhibitory effects of affinity-purified anti-Rpf antibodies on growth of the Academia strain of *M. tuberculosis* and *M. bovis* Bacillus Calmette-Guérin *in vitro* (Mukamolova *et al*., 2002a). However, during the course of this study, the construction of a mutant of *Corynebacterium glutamicum* lacking both *rpf*-like genes present in this organism was reported (Hartmann *et al*., 2004). The dispensability of the *rpf*-like genes in both *M. tuberculosis* and *C. glutamicum* thus suggests that *Mi. luteus*, which possesses a single, essential *rpf* (Mukamolova *et al*., 2002a), may represent an exception rather than the rule.

Deletion of the individual *rpfB-E* genes was previously shown to be accompanied by a modest upregulation of some or all of the remaining *rpf*-like genes (Downing *et al*., 2004). However, these subtle effects were reversed upon deletion of further *rpf*-like genes to a point at which the single *rpfB*, *rpfD* or *rpfE* gene remaining in the quadruple mutants was expressed at a level 1.3–1.8-fold lower than in the wild-type strain. These observations suggest that the remaining *rpf*-like genes do not compensate for loss of the other genes by transcriptional upregulation and argue against a significant degree of regulatory cross-talk within this gene family. Instead, the available data suggest the *rpf*-like genes are likely to be regulated by other, distinct mechanisms. In this respect, it is worth noting that *rpfA* has been shown to be subject to regulation by the cAMP receptor protein (Rickman *et al*., 2005), whereas *rpfC* is positively regulated by both the alternate sigma factor, SigD (Raman *et al*., 2004) and the site two protease homologue, Rv2869c (Makinoshima and Glickman, 2005).

A key *in vitro* phenotype associated with progressive *rpf*-like gene loss in *M. tuberculosis* is the inability to resuscitate spontaneously from a ‘non-culturable’ state. Like their progenitor triple mutant (Downing *et al*., 2005), both quadruple mutants assessed in this model displayed this phenotype. Significantly, the resuscitation defects were partly reversed by genetic complementation and/or by the addition of culture filtrate (an exogenous source of Rpfs). Therefore, the failure of the multiple mutants to resuscitate cannot simply be ascribed to poor survival of these strains in the Sauton's medium/sealed flask starvation model, but is attributable, at least in part, to a deficiency in Rpf function.

Progressive *rpf*-like gene loss also differentially affected the colony-forming ability of *M. tuberculosis* on agar-solidified media. Whereas *rpfB* or *rpfE* alone was sufficient to support a normal rate of colony formation, the mutant retaining only *rpfD* and its quintuple mutant derivative were impaired in this regard. Although the precise reason(s) why mutant cells grown on agar plates showed delayed colony formation are not known, we may speculate that this is a result of sudden exposure to stress (e.g. elevated oxygen concentration, high surface tension). In *Mi. luteus*, which contains a single essential *rpf* gene, Rpf function is vital for survival under conditions that are inappropriate for bacterial growth, or when cells are exposed to stresses such as nutrient starvation (reviewed by Mukamolova *et al*., 2003). In *M. tuberculosis*, which contains five nonessential *rpf*-like genes, the phenotype observed *in vitro* (delayed colony formation when transferred from a liquid to a solid medium) is less pronounced, although evident for mutant cells even in the absence of any stress. However, the greater challenge posed by prolonged starvation in stationary phase *in vitro* resulted in failure to grow on plates for both the wild-type and the mutant strains (Table 3). Significantly, the same mutants that displayed delayed colony formation on 7H11 agar were also most sensitive to SDS. Together, the data therefore suggest that Rpf deficiency results in a cell wall defect that renders *M. tuberculosis* hypersensitive to stresses that affect the cell envelope, with the effect being most pronounced in cells deficient in RpfB and RpfE.

The ability of individual *rpf*-like genes to complement the delayed colony formation phenotype of the quintuple mutant provided a direct means of differentiating function and/or potency within the RpfA-E family. Importantly, the notion of a ‘functional hierarchy’ within this family inferred from the *in vitro* data (Fig. 2) was further supported by the *in vivo* data demonstrating that loss of *rpfD* from the double mutant, ΔAC, had a lesser attenuating effect than loss of *rpfB* on growth in mouse lung (Downing *et al*., 2005) and loss of *rpfD* from the triple mutant, ΔACB, had a less pronounced effect than loss of *rpfE* on persistence (this work). These observations therefore suggest that RpfB and RpfE rank above RpfD and RpfC in the functional hierarchy. It is particularly interesting to note that the Rpfs that rank highest in this hierarchy – RpfB and RpfE – correspond to the two Rpfs shown to interact with the partnering peptidoglycan hydrolase, RipA (Hett *et al*., 2007).

The quadruple mutants, ΔACBD and ΔACBE, were severely impaired for growth and persistence in mice. The single (ΔA) and triple mutant (ΔACB) progenitors of these strains were assessed for virulence in a different mouse model (C57BL/6JCit) (Downing *et al*., 2005) from that employed in the present study (B6D2/F_1_), thus precluding a direct comparison of the datasets. The data nonetheless suggested that progressive *rpf*-like gene loss was accompanied by progressive attenuation for growth *in vivo*, with loss of *rpfE* or *rpfB* being more attenuating than loss of *rpfD* (Downing *et al*., 2005). However, attempts to complement the *in vivo* phenotype of ΔACBE yielded equivocal results: during early infection, a partial restoration of virulence was observed, but the effect was lost at later stages. The reasons underlying the variation observed between animals and why the partial restoration of virulence was not sustained are unclear, but may include: (i) incomplete/inadequate restoration of *rpf*-like gene function and/or temporal differences in expression of the complementing genes due to the limitations inherent in the complementation methodology (integration, at a different chromosomal location (*attB*), of the complementing gene(s) with varying lengths of flanking sequence), (ii) secondary effects caused by genetic elements carried on the fragments cloned in the complementation vector, (iii) instability/loss/rearrangement of the integration vector, and (iv) the accumulation of attenuating second-site mutations through the repeated cycles of allelic exchange mutagenesis required to produce ΔACBE and the other multiple mutant strains. Attenuation through loss of PDIM production was ruled out as a contributing factor, as all strains – including the parental wild-type – were equally poor producers of this lipid. However, the possibility that other attenuating second-site mutations were acquired during the construction and passage of the mutant strains cannot be excluded. Therefore, the attenuation observed *in vivo* cannot, as yet, be attributed exclusively and unequivocally to *rpf*-like gene loss. This study has highlighted the difficulties in correlating genotype with phenotype in multiple mutant strains of *M. tuberculosis*. In this particular case, pair-wise comparisons of growth and survival in primary mouse macrophages, which may be a more relevant *ex vivo* model system than human PBMCs, and of virulence in a single mouse model for the mutant strains ranging from ΔAC to ΔACBED or ΔACDEB, alongside their complemented counterparts, are planned in order to address this issue systematically.

In conclusion, the results described herein have confirmed the collective dispensability of *rpfA-E* for growth of *M. tuberculosis* in broth culture, and have suggested a functional hierarchy within this gene family under the conditions tested. We have yet to unravel the complexities that underpin the *in vivo* phenotypes and relate them to the various *in vitro* phenotypes associated with *rpf*-like gene loss. The fact that some Rpfs interact with other proteins in the cell to form protein complexes that may cleave distinct forms of peptidoglycan (Hett *et al*., 2007) further adds to the complexity of Rpf function and regulation. However, the collection of mutant strains reported in this and earlier studies (Downing *et al*., 2004; 2005) have provided an important resource for future biochemical, microbiological and physiological studies on this fascinating family of proteins.

## Experimental procedures

### Bacterial strains and culture conditions

The bacterial strains and plasmids used in this study are detailed in Table 2. *E. coli* strains were grown in Luria–Bertani broth (LB) or on Luria agar. Unless otherwise indicated, *M. tuberculosis* strains were grown in Middlebrook 7H9 media (Merck) supplemented with 0.2% glycerol, Middlebrook oleic acid-albumin-dextrose-catalase (OADC) enrichment (Merck) and 0.05% Tween 80. Ampicillin (Ap), kanamycin (Km) and gentamicin (Gm) were used in *E. coli* cultures at final concentrations of 100, 50 and 10 μg ml^−1^ respectively; hygromycin (Hyg) and Km were used in *M. tuberculosis* cultures at final concentrations of 50 and 25 μg ml^−1^ respectively.

### Allelic exchange mutagenesis

Allelic exchange mutagenesis was carried out by previously described methods (Parish and Stoker, 2000; Gordhan and Parish, 2001; Downing *et al*., 2004; 2005) using the knockout vectors pRPFBΔ2, pRPFDΔ2 and pRPFEΔ2, which harbour in-frame deletions in the *rpfB*, *rpfD* and *rpfE* genes respectively (Downing *et al*., 2004). The quadruple mutants, ΔACBD and ΔACBE, were produced from the triple mutant progenitor, ΔACB (KDT8) (Downing *et al*., 2005) whereas ΔACDE was produced from the triple mutant, ΔACD (KDT9) (Downing *et al*., 2005) (Table 2). ΔACBE was the first quadruple mutant obtained and was thus used to generate the quintuple mutant, ΔACBED, by deletion of *rpfD*. Mutant genotypes were confirmed by Southern hybridization using the previously described restriction enzymes and probes (Downing *et al*., 2004) to analyse the *rpfA-E* loci in each strain (Fig. S1). In the construction of the quintuple mutant, three independent double cross-over recombinants were recovered from the two-step selection procedure (Parish and Stoker, 2000). Southern blot analysis confirmed that all three isolates carried the expected deletion allele in *rpfD* in addition to the deletion alleles in *rpfA*, *rpfB*, *rpfC* and *rpfE* genes present in the parental strain (Fig. S1, and not shown). After ΔACDE was obtained, this strain was used to construct another quintuple mutant, ΔACDEB, by deletion of *rpfB*. All quadruple and quintuple mutant strains obtained were screened by PCR amplification at all five loci using the primers described in Table S1 which were designed to differentiate the wild-type and Δ*rpfA-E* deletion alleles. In all cases, PCR products of the expected sizes were obtained (Fig. S2), confirming the mutant genotypes. As the three isolates of the ΔACBED mutant were genotypically indistinguishable from one another, one was arbitrarily selected for phenotypic characterization.

### Construction of complementation vectors

A 1824 bp XhoI-HindIII fragment from pGS3RPF containing the 672 bp *rpf* gene from *Mi. luteus* with 430 bp of native upstream sequence, and cloned downstream of the *hsp60* promoter from pSMT3 was transferred to pHINT (O'Gaora *et al*., 1997) to yield pMLUWP. A 4249 bp HindIII/Asp718 fragment from cosmid G7A (Brosch *et al*., 1998) containing *rpfC* was cloned in pEM75 to yield pEMRPFC. A 2879 bp EcoRI/BglII fragment from pEMRPFC containing the *fbpB*, Rv1885c and *rpfC* genes preceded by 631 bp of sequence upstream of *fbpB* was cloned in pHINT to produce pHRPFC. To produce complementation vectors carrying *rpfD* and/or *rpfE*, these genes and flanking sequences were PCR-amplified from chromosomal DNA and cloned in pBluescriptKS+ to yield pBRPFD and pBRPFE respectively (see Table S1 for a description of the primer sequences and amplicon properties). A 1640 bp SpeI fragment was excised from pBRPFD and cloned in pHRPFC to produce pHRPFCD, whereafter a 1164 bp BamHI fragment from pBRPFE was cloned in pHRPFCD to create pHRPFCDE. A 3027 bp XhoI fragment from cosmid G9D (Brosch *et al*., 1998) containing the *rpfB* gene was cloned in pEM75 to create pEMRPFB. A 1958 bp ClaI fragment from this vector containing the *rpfB* gene and 640 bp of upstream sequence was cloned in pHRPFC to yield pHRPFCB into which the *rpfD* gene from pBRPFD was cloned to create pHRPFCBD. The complementation vectors were delivered into the mutant host strains by electroporation, and integrants were selected on Hyg-containing media. However, expression analysis by RT-PCR revealed that *rpfB* was not expressed in strains carrying the latter two constructs. Therefore, a second *rpfB*-containing cassette was constructed by cloning a 6152 bp BamHI-HindIII fragment from cosmid G9D to create pEMRPFB2. A 1904 bp SacI fragment containing the *rpfB* gene was cloned from pEMRPFB2 into pMV306H, a derivative of pMV306 (Stover *et al*., 1991), to yield pMRPFB. For complementation with the other individual *rpf*-like genes, a 1925 bp SpeI fragment containing *rpfD* was cloned into pMV306H to yield pMRPFD and the 1164 bp BamHI fragment from pBRPFE containing *rpfE* was cloned into pMV306H to yield pMRPFE. Plasmid maps of the complementation vectors are shown in Fig. S3. Complementation vectors carrying the individual *rpf*-like genes were electroporated into ΔACBED and ΔACBE and Hyg-resistant (Hyg^R^) transformants were picked and screened for expression of corresponding gene by RT-PCR using the primers described below.

### Gene expression analysis by real-time, qRT-PCR

To monitor the expression of *rpf*-like genes in the mutant strains and complemented derivatives, RNA was extracted from mid-logarithmic phase cultures (OD_600_ = 0.8–1.0), using previously described methods (Downing *et al*., 2004). Real-time, qRT-PCR analysis of expression of the *rpfB*, *rpfC*, *rpfD*, *rpfE* and *sigA* genes in the wild-type and mutant strains was performed using the primer sets described by Downing *et al*. (2004). Synthesis of cDNA was carried out at 60°C for 30 min using 500 ng of RNA in a 20 μl reaction mixture containing 1× PCR buffer without MgCl_2_ (Roche), 12.5 nM reverse primers, 4 mM MgCl_2_, 0.8 mM dNTPs, 3% DMSO and 1 μl of Enhanced Avian Reverse Transcriptase (Sigma). For qRT-PCR, 2 μl of cDNA was used for amplification with LightCycler FastStart DNA Master SYBR Green I kit in the Roche LightCycler (version 1.5). A standard curve based on 10-fold serial dilutions of H37Rv genomic DNA was established for each gene assayed and a linear or polynomial equation was modelled on to the amplification data using the LightCycler software (version 4.0) to determine transcript levels in RNA samples. Absolute numbers of transcript were normalized to the number of *sigA* transcripts in the same sample and the normalized data were compared with normalized transcript levels in the H37Rv control. The analysis was performed in triplicate biological samples, each in duplicate. Expression of the *rpfB-E* genes in the mutant strains and their genetically complemented counterparts was also analysed by RT-PCR using the *rpfB*, *rpfD* and *rpfE* primers described by Tufariello *et al*. (2004), the *rpfC* primers described by Downing *et al*. (2004) and the *sigA* primers described by Dawes *et al*. (2003). Briefly, cDNA synthesis was carried out as described above and 2 μl of cDNA or genomic DNA standard was used for PCR amplification in an Eppendorf MasterCycler. Products were visualized on a 2% agarose gel. Expression of the *Mi. luteus rpf* gene was similarly monitored by semiquantitative RT-PCR using the primers described in Table S1.

### Analysis of PDIM production in *M. tuberculosis*

Cultures (10 ml) were grown to an OD_600_ = 0.5 in Middlebrook 7H9 media supplemented with OADC and 0.05% Tween before the addition of 2 μCi of [^14^C]-propionic acid (Sigma) and growth for a further 24–48 h. The culture was then harvested and resuspended in 5 ml of 10:1 methanol : 0.3% NaCl before adding 5 ml of petroleum ether. The mixture was vortexed (5 min) and centrifuged (550 *g,* 10 min) before removing the top layer and re-extracting the bottom layer with petroleum ether. The two extracted fractions were pooled, mixed with 10 ml of chloroform and incubated overnight to allow for solvent evaporation. An aliquot (24–30 μl) of the lipid extract was spotted onto Silica gel 60F254 thin-layer chromatography plates which were developed in 9:1 petroleum ether : diethyl ether. Labelled lipids were visualized autoradiographically by exposing the dried plates to Kodak Biomax imaging film at room temperature for 10–12 days.

### Growth of mutant strains in broth culture

To assess the growth of the mutant strains in axenic culture, 50 ml cultures were grown standing in 550 ml tissue culture flasks containing Middlebrook 7H9 broth for a period of four weeks. Growth was monitored by enumerating cfu from samples drawn at various time points, which were serially diluted and plated on Middlebrook 7H10 agar. The ability of the mutant strains to form colonies on agar-solidified Middlebrook 7H10 or 7H11 medium was also assessed by plating serial dilutions of logarithmic phase cultures (OD_600_ = 0.7–1.0) and colony formation was scored after 18 days for all strains and again at 34 days for strains that displayed delayed colony formation.

### SDS susceptibility testing

Log-phase cultures (OD_600_ = 0.6) were serially diluted in Middlebrook 7H9 broth, and 10 μl of neat, 10-fold and 100-fold diluted cultures were spotted onto plates containing 7H11 agar or 7H11 agar supplemented with 0.1%, 0.01% or 0.001% SDS. Plates were incubated at 37°C for 10 days before scoring for growth.

### Resuscitation of ‘non-culturable’ cells

Strains were exposed to 3.5 months' nutrient starvation without oxygen input under the previously described Sauton's medium/sealed flask model in order to render the bacteria ‘non-culturable’, i.e. unable to form colonies (Downing *et al*., 2005). To assess the ability of ‘non-culturable’ cells of the various strains to resuscitate spontaneously, resuscitation and MPN assays were carried out as previously described (Downing *et al*., 2005) using either Sauton's medium supplemented with ADC, with or without further supplementation with 50% culture filtrate from a late-logarithmic-phase culture of wild-type *M. tuberculosis*. The tubes were incubated at 37°C without agitation for 2 months before recording the number of tubes with visible growth and calculating the MPN values (de Man, 1975).

### Growth in macrophages

Human PBMCs were isolated and immediately infected with wild-type or mutant strains of *M. tuberculosis* at a multiplication of infection of 1 bacillus per 1 monocyte (1:1) in R20, as previously described (Manca *et al*., 1999). Bacterial cfu were assessed daily for 4 days using previously reported methods (Post *et al*., 2001).

### Aerosol infection of mice

Eight- to ten-week-old female B6D2/F_1_ mice, free of common viral pathogens from Charles River Laboratories (Wilmington, MA) or Jackson Laboratories (Bar Harbor, ME), were infected with the H37Rv, ΔACBD, ΔACBE and ΔACBE + CDE strains through the respiratory route, as previously described (Manca *et al*., 1999). Mice were infected by exposure to aerosols containing *M. tuberculosis* using a nose only exposure system (In Tox Products, Albuquerque, MN). In most cases, approximately 200–800 organisms were implanted in the lungs of each mouse, as confirmed by plating lung homogenates 3 h after infection. Bacterial loads (cfu) in the lungs and spleen of infected mice were assessed at selected time points over a period of 240 days. For the infections with H37Rv, ΔACBD and ΔACBE, groups of 10 infected mice were set aside to monitor survival.

### Statistics

The independent Student's *t*-test or paired *t*-test was used to assess statistical significance of pair-wise comparisons using GraphPad Prism Software (http://www.graphpad.com/quickcalcs/ttest1.cfm).

## Supplementary material

This material is available as part of the online article from:

http://www.blackwell-synergy.com/doi/abs/10.1111/j.1365-2958.2007.06078.x

(This link will take you to the article abstract).

## References

[b1] Brosch R, Gordon SV, Billault A, Garnier T, Eiglmeier K, Soravito C (1998). Use of a *Mycobacterium tuberculosis* H37Rv bacterial artificial chromosome library for genome mapping, sequencing, and comparative genomics. Infect Immun.

[b2] Camacho LR, Ensergueix D, Perez E, Gicquel B, Guilhot C (1999). Identification of a virulence gene cluster of *Mycobacterium tuberculosis* by signature-tagged transposon mutagenesis. Mol Microbiol.

[b3] Cohen-Gonsaud M, Barthe P, Bagneris C, Henderson B, Ward J, Roumestand C, Keep NH (2005). The structure of a resuscitation-promoting factor domain from *Mycobacterium tuberculosis* shows homology to lysozymes. Nat Struct Mol Biol.

[b4] Corbett EL, Watt CJ, Walker N, Maher D, Williams BG, Raviglione MC, Dye C (2003). The growing burden of tuberculosis: global trends and interactions with the HIV epidemic. Arch Intern Med.

[b5] Cox JS, Chen B, McNeil M, Jacobs WR (1999). Complex lipid determines tissue-specific replication of *Mycobacterium tuberculosis* in mice. Nature.

[b6] Dawes SS, Warner DF, Tsenova L, Timm J, McKinney JD, Kaplan G, Mizrahi V (2003). Ribonucleotide reduction in *Mycobacterium tuberculosis*: function and expression of genes encoding class Ib and class II ribonucleotide reuctases. Infect Immun.

[b7] Domenech P, Reed MB, Barry CE (2005). Contribution of the *Mycobacterium tuberculosis* MmpL protein family to virulence and drug resistance. Infect Immun.

[b8] Downing KJ, Betts JC, Young DI, McAdam RA, Kelly F, Young M, Mizrahi V (2004). Global expression profiling of strains harbouring null mutations reveals that the five *rpf*-like genes of *Mycobacterium tuberculosis* show functional redundancy. Tuberculosis (Edinb).

[b9] Downing KJ, Mischenko VV, Shleeva MO, Young DI, Young M, Kaprelyants AS (2005). Mutants of *Mycobacterium tuberculosis* lacking three of the five *rpf*-like genes are defective for growth *in vivo* and for resuscitation *in vitro*. Infect Immun.

[b10] Dye C, Williams BG, Espinal MA, Raviglione MC (2002). Erasing the world's slow stain: strategies to beat multidrug-resistant tuberculosis. Science.

[b11] Fenhalls G, Stevens L, Moses L, Bezuidenhout J, Betts JC, van Helden P (2002). *In situ* detection of *Mycobacterium tuberculosis* transcripts in human lung granulomas reveals differential gene expression in necrotic lesions. Infect Immun.

[b12] Gillespie J, Barton LL, Rypka EW (1986). Phenotypic changes in mycobacteria grown in oxygen-limited conditions. J Med Microbiol.

[b13] Gordhan BG, Parish T (2001). Gene replacement using pre-treated DNA. Methods Mol Med.

[b14] Hartmann M, Barsch A, Niehaus K, Puhler A, Tauch A, Kalinowski J (2004). The glycosylated cell surface protein Rpf2, containing a resuscitation-promoting factor motif, is involved in intercellular communication of *Corynebacterium glutamicum*. Arch Microbiol.

[b15] Hett EC, Chao MC, Steyn AJ, Fortune SM, Deng LL, Rubin EJ (2007). A partner for the resuscitation-promoting factors of *Mycobacterium tuberculosis*. Mol Microbiol.

[b16] Keep NH, Ward JM, Cohen-Gonsaud M, Henderson B (2006). Wake up! Peptidoglycan lysis and bacterial non-growth states. Trends Microbiol.

[b17] Kell DB, Young M (2000). Bacterial dormancy and culturability: the role of autocrine growth factors. Curr Opin Microbiol.

[b18] Makinoshima H, Glickman MS (2005). Regulation of *Mycobacterium tuberculosis* cell envelope composition and virulence by intramembrane proteolysis. Nature.

[b19] de Man JC (1975). The probability of most probable numbers. Eur J Appl Microbiol.

[b20] Manca C, Paul S, Barry CE, Freedman VH, Kaplan G (1999). *Mycobacterium tuberculosis* catalase and peroxidase activities and resistance to oxidative killing in human monocytes *in vitro*. Infect Immun.

[b21] Manjunatha UH, Boshoff H, Dowd CS, Zhang L, Albert TJ, Norton JE (2006). Identification of a nitroimidazo-oxazine-specific protein involved in PA-824 resistance in *Mycobacterium tuberculosis*. Proc Natl Acad Sci USA.

[b22] Mukamolova GV, Kaprelyants AS, Young DI, Young M, Kell DB (1998). A bacterial cytokine. Proc Natl Acad Sci USA.

[b23] Mukamolova GV, Kaprelyants AS, Kell DB, Young M (2003). Adoption of the transiently non-culturable state – a bacterial survival strategy?. Adv Microb Physiol.

[b24] Mukamolova GV, Turapov OA, Kazarian K, Telkov M, Kaprelyants AS, Kell DB, Young M (2002a). The *rpf* gene of *Micrococcus luteus* encodes an essential secreted growth factor. Mol Microbiol.

[b25] Mukamolova GV, Turapov OA, Young DI, Kaprelyants AS, Kell DB, Young M (2002b). A family of autocrine growth factors in *Mycobacterium tuberculosis*. Mol Microbiol.

[b26] Mukamolova GV, Murzin AG, Salina EG, Demina GR, Kell DB, Kaprelyants AS, Young M (2006). Muralytic activity of *Micrococcus luteus* Rpf and its relationship to physiological activity in promoting bacterial growth and resuscitation. Mol Microbiol.

[b27] O'Gaora P, Barnini S, Hayward C, Filley E, Rook G, Young DB, Thole J (1997). Mycobacteria as immunogens. Development of expression vectors for use in multiple species. Med Princ Pract.

[b28] Parish T, Stoker NG (2000). Use of a flexible cassette method to generate a double unmarked *Mycobacterium tuberculosis tlyA plcABC* mutant by gene replacement. Microbiology.

[b29] Post FA, Manca C, Neyrolles O, Ryffel B, Young DB, Kaplan G (2001). *Mycobacterium tuberculosis* 19-kilodalton lipoprotein inhibits *Mycobacterium smegmatis*-induced cytokine production by human macrophages in vitro. Infect Immun.

[b30] Rachman H, Strong M, Ulrichs T, Grode L, Schuchhardt J, Mollenkopf H (2006). Unique transcriptome signature of *Mycobacterium tuberculosis* in pulmonary tuberculosis. Infect Immun.

[b31] Raman S, Hazra R, Dascher CC, Husson RN (2004). Transcription regulation by the *Mycobacterium tuberculosis* alternative sigma factor SigD and its role in virulence. J Bacteriol.

[b32] Ravagnani A, Finan CL, Young M (2005). A novel firmicute protein family related to the actinobacterial resuscitation-promoting factors by non-orthologous domain displacement. BMC Genomics.

[b33] Rickman L, Scott C, Hunt DM, Hutchinson T, Menendez MC, Whalan R (2005). A member of the cAMP receptor protein family of transcription regulators in *Mycobacterium tuberculosis* is required for virulence in mice and controls transcription of the *rpfA* gene coding for a resuscitation promoting factor. Mol Microbiol.

[b34] Rousseau C, Winter N, Pivert E, Bordat Y, Neyrolles O, Ave P (2004). Production of phthiocerol dimycocerosates protects *Mycobacterium tuberculosis* from the cidal activity of reactive nitrogen intermediates produced by macrophages and modulates the early immune response to infection. Cell Microbiol.

[b35] Shleeva MO, Bagramyan K, Telkov MV, Mukamolova GV, Young M, Kell DB, Kaprelyants AS (2002). Formation and resuscitation of ‘non-culturable’ cells of *Rhodococcus rhodochrous* and *Mycobacterium tuberculosis* in prolonged stationary phase. Microbiology.

[b36] Stewart GR, Robertson BD, Young DB (2003). Tuberculosis: a problem with persistence. Nat Rev Microbiol.

[b37] Stover CK, de la Cruz VF, Fuerst TR, Burlein JE, Benson LA, Bennett LT (1991). New use of BCG for recombinant vaccines. Nature.

[b38] Tufariello JM, Jacobs WR, Chan J (2004). Individual *Mycobacterium tuberculosis* resuscitation-promoting factor homologues are dispensable for growth *in vitro* and *in vivo*. Infect Immun.

[b39] Tufariello JM, Mi K, Xu J, Manabe YC, Kesavan AK, Drumm J (2006). Deletion of the *Mycobacterium tuberculosis* resuscitation-promoting factor Rv1009 gene results in delayed reactivation from chronic tuberculosis. Infect Immun.

[b40] Young M, Mukamolova GV, Kaprelyants AS, Parish T (2005). Mycobacterial dormancy and its relation to persistence. Mycobacterium: Molecular Microbiology.

