# Supplementary material for: The resuscitation-promoting factors of Mycobacterium tuberculosis are required for virulence and resuscitation from dormancy but are collectively dispensable for growth in vitro
Source: Mol Microbiol. 2007 Dec 21;67(3):672–84. doi: 10.1111/j.1365-2958.2007.06078.x (PMC2229633; doi:10.1111/j.1365-2958.2007.06078.x)
Supplement: Supplementary file 1 [file mmi0067-0672-SD1.pdf]

SUPPLEMENTARY INFORMATION

The resuscitation-promoting factors of *Mycobacterium tuberculosis* are required for virulence and resuscitation from dormancy but are collectively dispensable for growth in vitro

Bavesh D. Kana[1*] Bhavna G. Gordhan[1], Katrina J. Downing[1], Nackmoon Sung[2¶], Galina Vostroktunova[3], Edith E. Machowski[1], Liana Tsenova[2], Michael Young[4], Arseny Kaprelyants[3], Gilla Kaplan[2], Valerie Mizrahi[1]*

[1] MRC/NHLS/WITS Molecular Mycobacteriology Research Unit, DST/NRF Centre of Excellence for Biomedical TB Research, School of Pathology, University of the Witwatersrand and the National Health Laboratory Service, Johannesburg 2000, South Africa

[2] Laboratory of Mycobacterial Immunity and Pathogenesis, Public Health Research Institute, International Center for Public Health, 225 Warren St., Newark, NJ 07103-3535, USA

[3] Bakh Institute of Biochemistry, 117071 Moscow, Russia

[4] Institute of Biological Sciences, Aberystwyth University, Wales, UK

Table S1. Oligonucleotides used in this study

Name	Sequence (5’-3’)[a]	Application	Amplicon properties/ reference
RpfDF	GGGCCACTAGTCCTCTAGATGTTTTCG ATGCGCTCC	Forward primer used to amplify *rpfD* genomic region	1656 bp amplicon containing 553bp of sequence upstream of Rv2390, the entire Rv2390 gene, 3 bp of intervening sequence, the entire *rpfD* gene and 39 bp of sequence downstream of *rpfD*
RpfDR	GGGCGACTAGTACCAGCTAGGGTGGC CAGC	Reverse primer used to amplify *rpfD* genomic region	
RpfEF	CGCCGGATCCCTCTAGAATCACAGTG CGGCACTTAG	Forward primer used to amplify *rpfE* genomic region	1180 bp amplicon containing the divergently transcribed Rv2451 gene upstream of *rpfE*, 82 bp of intervening sequence, the entire *rpfE* gene and 40 bp of sequence downstream of *rpfE*
RpfER	CGGCGGATCCGACCGGATTTGGGAGC	Reverse primer used to amplify *rpfE* genomic region	
RpfA-F1	ATAGCACGCCGAACTCCATC		
RpfA-R1	GTGGCGGAGGTAGCTGAAGA	Forward, reverse and wild type primers used for PCR genotyping of *rpfA* and Δ*rpfA* alleles	371 bp amplicon from wild type *rpfA* allele using RpfA-F1/ RpfA-R1 pair, and 577 bp amplicon from Δ*rpfA* allele using RpfA-F1/ RpfA-W pair
RpfA-W	GCCACCTGATCCCATTCC		
RpfB-F1	TTATGGGCTAGGGTGGATGC		
RpfB-R1	GCAGCCGAGAAGCCAGTAGT	Forward, reverse and wild type specific primers used for PCR genotyping of *rpfB* and Δ*rpfB* alleles	394 bp amplicon from wild type *rpfB* allele using RpfB-F1/ RpfB-R1 pair and 565 bp amplicon from Δ*rpfB* allele using RpfB-F1/ RpfB-W pair
RpfB-W	CCCGTTCTCTTCGACGATGT		
RpfC-F1	CTCTATCAACGGGCCCTGAC		
RpfC-R1	CACAGCAAACCCGAACTCAC	Forward, reverse and wild type primers used for PCR genotyping of *rpfC* and Δ*rpfC* alleles	371 bp amplicon from wild type *rpfC* allele using RpfC-F1/ RpfC-R1 pair and 556 bp amplicon from mutant Δ*rpfC* allele using RpfC-F1/ RpfC-W pair

RpfC-W	GAACTGCAGTCCGCCGTATT		RpfC-F1/ RpfC-W pair
RpfD-F1	CGGATCGACCACAACATGAG		
RpfD-R1	GCCGGGGTGTAGGTATTGAA	Forward, reverse and wild type primers used for PCR genotyping of *rpfD* and Δ*rpfD* alleles	375 bp amplicon from wild type *rpfD* allele using RpfD-F1/ RpfD-R1 pair and 538 bp amplicon from Δ*rpfD* allele using RpfD-F1/ RpfD-W pair
RpfD-W	ACACCGTGCATACGATCCTG		
RpfE-F1	TTATCGTACGGTCCCCTTGG		
RpfE-R1	TCAGGATCGGCCAGGTCT	Forward, reverse and wild type primers for PCR genotyping of *rpfE* and Δ*rpfE* alleles	357 bp amplicon from wild type *rpfE* allele using RpfE-F1/ RpfE-R1 pair and 575 bp amplicon from Δ*rpfE* allele using RpfE-F1/ RpfE-W pair
RpfE-W	CGTCGGCATTGGCGATAC		
MluF	CACCTGGGACATCAACACC	Forward primer used for RT-PCR analysis of *Mi. luteus rpf*	N/A
MluR	TTGATCTGCTCGGCCTTC	Reverse primer used for RT- PCR analysis of *Mi. luteus rpf*	

a. Restriction enzyme sites engineered for cloning are underlined

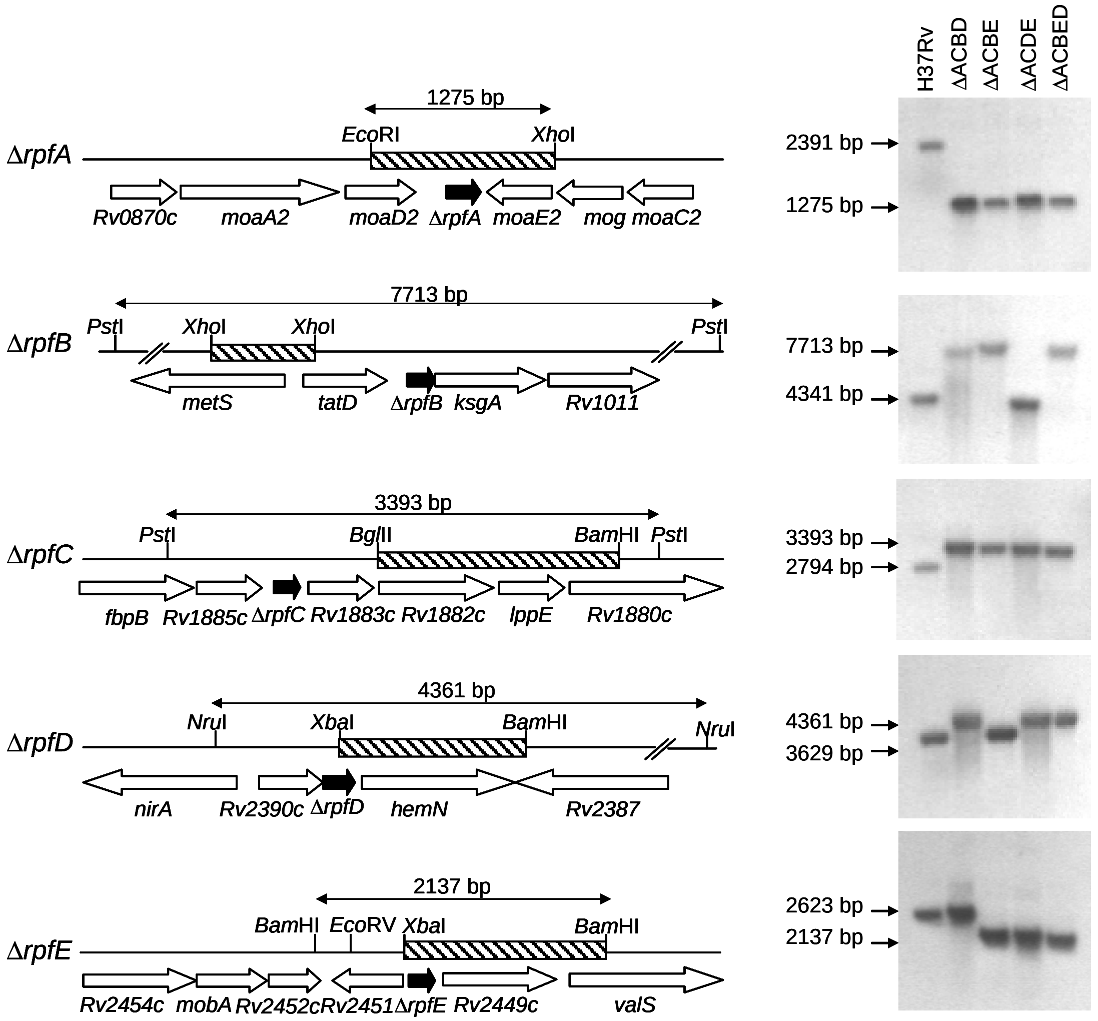

Figure S1. Genotypic characterization of the quadruple and quintuple mutant strains by Southern blot analysis using previously described restriction enzymes and probes (Downing et al., 2004). The chromosomal loci of the deletion mutations are shown on the left and the corresponding Southern blots are shown on the right. The residual, deleted *rpf*-like genes are shown as solid black arrows and the probes as hatched boxes. Lane 1, H37Rv; lane 2, ΔACBD; lane 3, ΔACBE; lane 4, ΔACDE; lane 5, ΔACBED.

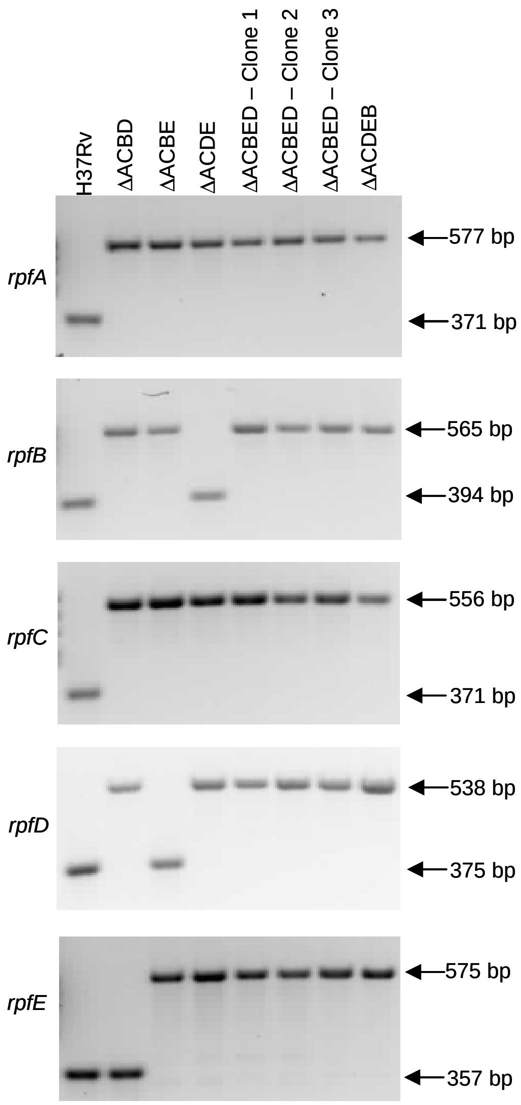

Figure S2. PCR-based genotyping of quadruple and quintuple mutant strains. Chromosomal DNA template was used to amplify the *rpfA-E* alleles from the wild type and mutant strains using the primer pairs described in Table S1. The expected sizes of the amplicons are as follows: *rpfA*, 371 bp; Δ*rpfA*, 577 bp; *rpfB*, 394 bp; Δ*rpfB*, 565 bp; *rpfC*, 371 bp; Δ*rpfC*, 556 bp; *rpfD*, 375 bp; Δ*rpfD*, 538 bp; *rpfE*, 357 bp; Δ*rpfE*, 575 bp.

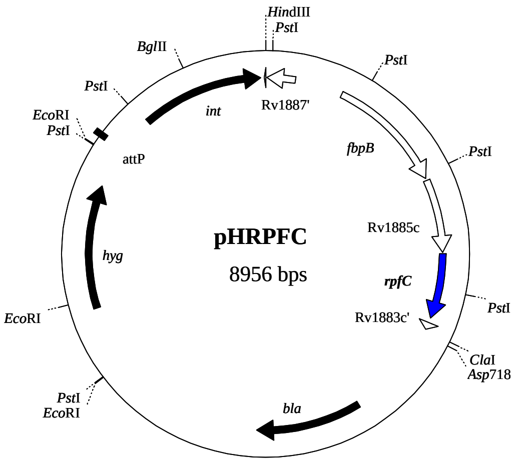
pHRPFC
8956 bps
HindIII
PstI
PstI
PstI
PstI
ClaI
Asp718
PstI
EcoRI
EcoRI
EcoRI
PstI
PstI
BglII
int
Rv1887'
fbpB
Rv1885c
rpfC
Rv1883c'
bla
hyg
attP

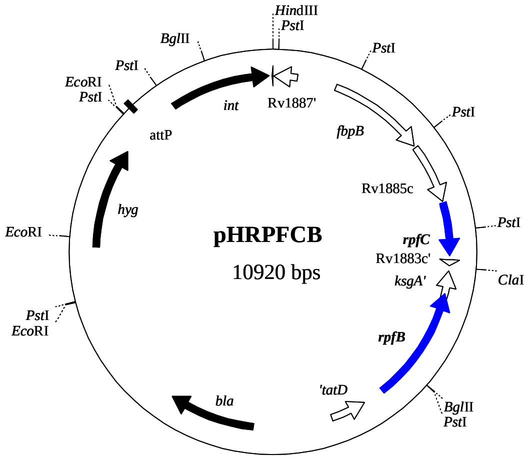
pHRPFCB
10920 bps
HindIII
PstI
PstI
PstI
PstI
ClaI
BglII
PstI
PstI
EcoRI
EcoRI
EcoRI
PstI
PstI
BglII
int
Rv1887'
fbpB
Rv1885c
rpfC
Rv1883c'
ksgA'
rpfB
'tatD
bla
hyg
attP

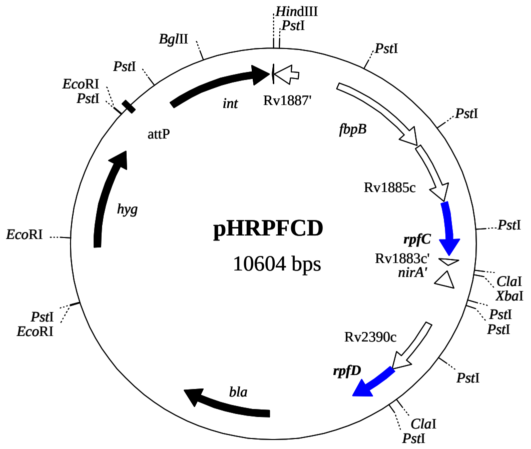
pHRPFCD
10604 bps
HindIII
PstI
PstI
PstI
PstI
ClaI
XbaI
PstI
PstI
PstI
ClaI
PstI
PstI
EcoRI
EcoRI
EcoRI
PstI
PstI
BglII
int
Rv1887'
fbpB
Rv1885c
rpfC
Rv1883c'
nirA'
Rv2390c
rpfD
bla
hyg
attP

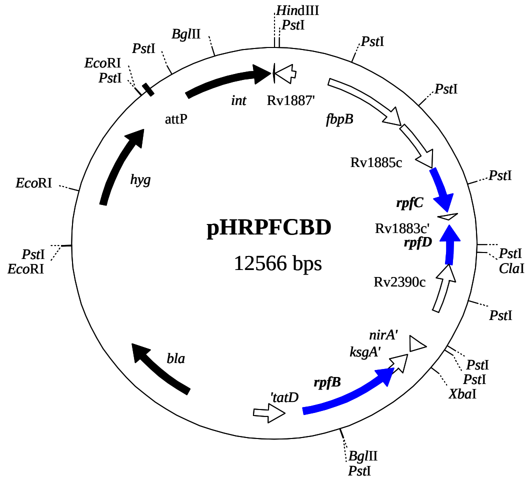
pHRPFCBD
12566 bps
HindIII
PstI
PstI
PstI
PstI
PstI
ClaI
PstI
PstI
PstI
XbaI
BglII
PstI
PstI
EcoRI
EcoRI
EcoRI
PstI
PstI
BglII
int
Rv1887'
fbpB
Rv1885c
rpfC
Rv1883c'
rpfD
Rv2390c
nirA'
ksgA'
rpfB
'tatD
bla
hyg
attP

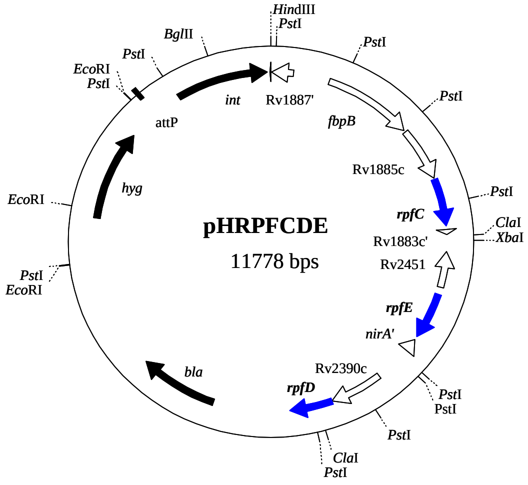
pHRPFCDE
11778 bps
HindIII
PstI
PstI
PstI
PstI
ClaI
XbaI
PstI
PstI
PstI
ClaI
PstI
PstI
EcoRI
EcoRI
EcoRI
PstI
PstI
BglII
int
Rv1887'
fbpB
Rv1885c
rpfC
Rv1883c'
Rv2451
rpfE
nirA'
Rv2390c
rpfD
bla
hyg
attP

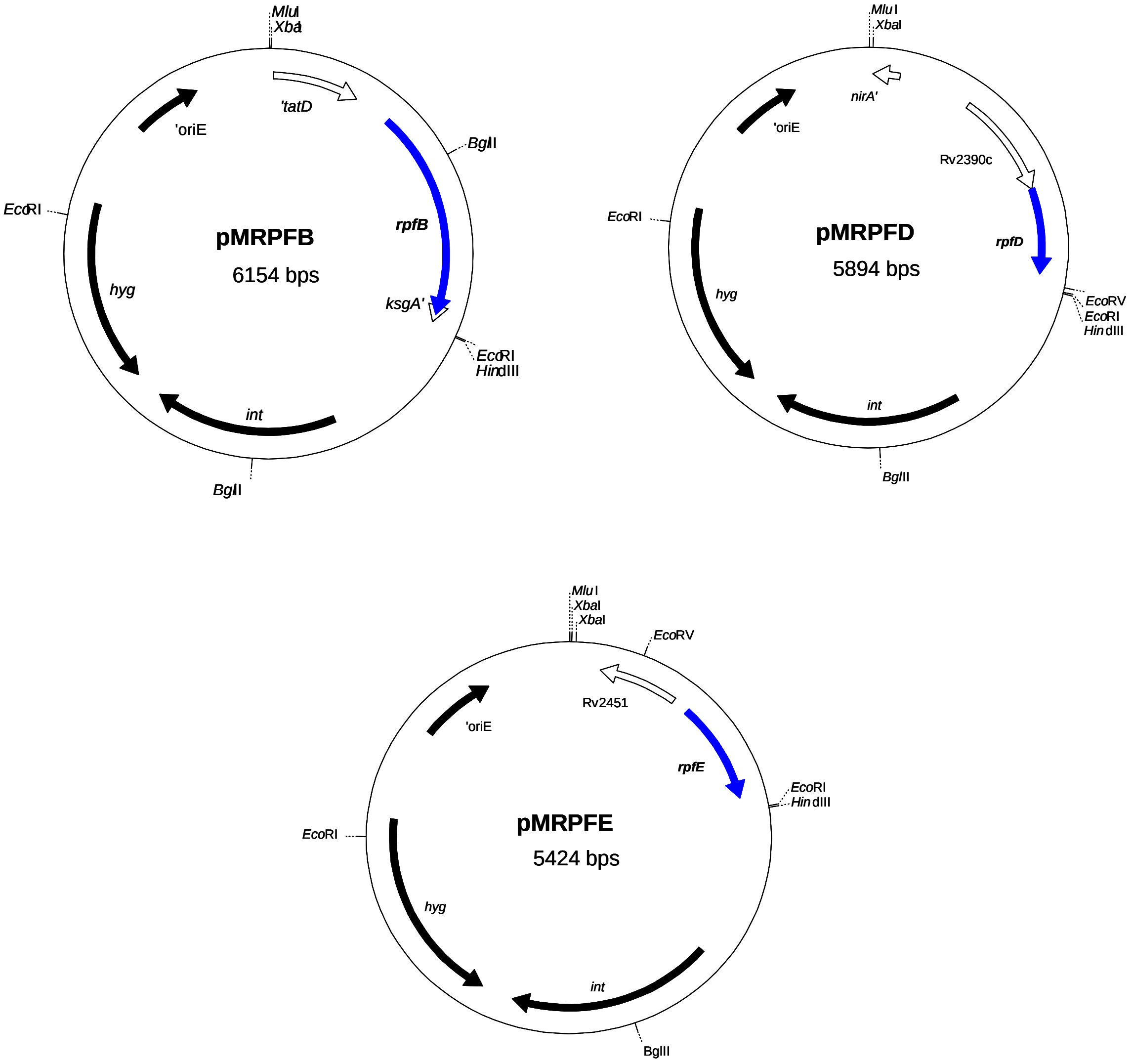

Figure S3. Plasmid maps of complementation vectors used in this study, as described in Table 1. The vectors on the first page of the figure are derivatives of the integration vector pHINT, whereas those on the second page are derivatives of pMV306H. *rpf*-like genes are shown as solid blue arrows, their flanking genes are shown as open arrows and relevant vector-derived genes are shown as sold black arrows.

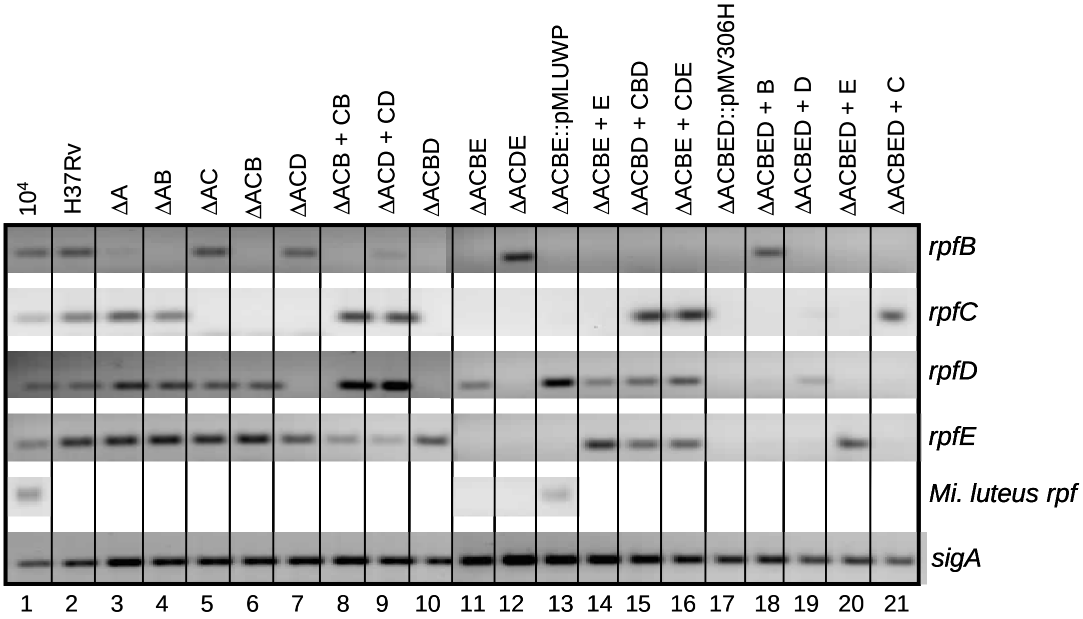

Figure S4. RT-PCR analysis of *rpf*-like gene expression in mutant strains and complemented counterparts. Lane 1, genomic DNA control (10^4 genome equivalents). All strains were assayed for expression of *rpfB*, *rpfC*, *rpfD*, *rpfE* and *sigA*, and ΔACBE and ΔACBE::pMLUWP were also assayed for expression of *Mi. luteus rpf*. The mutant strains are denoted by their genotypes (as described in Table 1), where "+" indicates genetic complementation with one or more *rpf*-like genes (denoted using single-letter nomenclature and as described in Table 1 and Fig. S3), empty vector (pMV306H) or vector carrying the *Mi. luteus rpf* (pMLUWP).

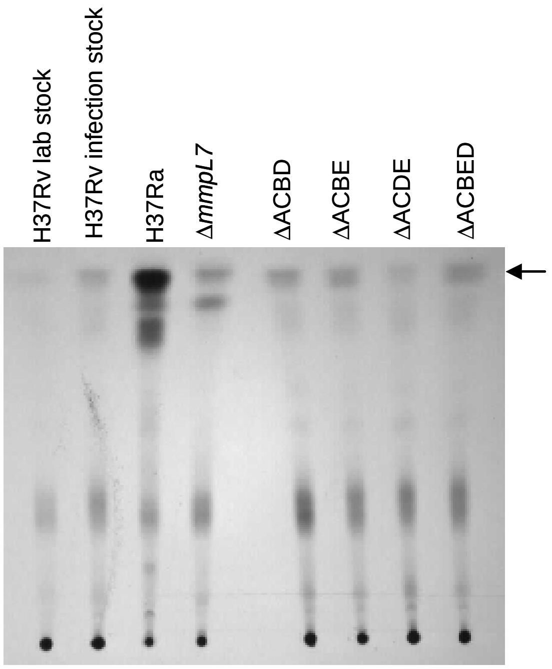

Figure S5. Comparative analysis of PDIM production by strains of MTB. The production of PDIM (indicated by the arrow) by the various strains was assessed by analyzing the incorporation, during growth, of [^{14}C]-propionate into lipid using thin-layer chromatography, as described under Materials and Methods.
